# If you blink at me, I’ll blink back. Domestic dogs’ feedback to conspecific visual cues

**DOI:** 10.1098/rsos.241703

**Published:** 2025-02-19

**Authors:** Chiara Canori, Tiziano Travain, Giulia Pedretti, Rachele Fontani, Paola Valsecchi

**Affiliations:** ^1^ Department of Medicine and Surgery, Via Gramsci 14, University of Parma, Parma 43126, Italy; ^2^ Department of Chemistry, Life Science and Environmental Sustainability, Viale delle Scienze 17/A, University of Parma, Parma 43124, Italy; ^3^ Department of Psychology, University of Warwick, Coventry, UK

**Keywords:** domestic dog, visual communication, HRV, nose lick, blink, appeasement behaviours

## Abstract

Blinking, along with other facial expressions, has been suggested to play a role in dogs’ intra- and interspecific communication, however the feedback this signal elicits from the audience is still poorly studied. In this study, we investigated the behavioural and physiological responses of 54 domestic dogs to videos of conspecifics performing blink. Based on existing literature, we hypothesized that dogs would show a higher rate of blinking when exposed to blink than to another facial expression (nose lick) and to an attentive still-looking face (control). Results showed that dogs blinked more during the blink video compared to the nose lick (NL) video, suggesting a mimicry phenomenon and implying a possible role of blinking in dogs’ communication. Cardiac analyses showed increased heart rate variability values during the video sessions independently to the type of facial signal projected, suggesting that the stimuli were not perceived as stressful. The present results open the door to future investigation of blink synchronization, as this aspect was not directly addressed in the present study. Future research should also explore the effects of eye blink and NL in modulating intraspecific social interactions.

## 1. Introduction

Communication is a process of information flow between a sender producing signals, and a receiver giving feedback [1–3]. Signals can be of different nature and convey different information about the sender: its identity, reproductive status, motivational and emotional states [1,4]. Communicating is essential for animals and visual signals play a pivotal role in many contexts such as group cohesion, conflict resolution and reconciliation [5–7]. Visual signals and facial expressions are present in most of the mammalian species and are well documented both in primates and canids (*non-human primates* [8–12]; *canids* [13–18]).

In wolves *Canis lupus*, some signals (e.g. licking the lips, flattening the ears, licking another’s mouth, softening the eyes) are thought to be used to prevent escalation of aggression between pack members and were termed ‘cut-off signals’ by Fox [19]. Given the common evolutionary history shared with wolves, many of these displays, including subtle signals such as nose licking and eye blinking [4,17,20–22], have been preserved in domestic dogs (*Canis familiaris*) and have been suggested to function as ‘appeasement signals’ aimed at advertising peaceful intentions [4,23,24]. A recent study found that eye blinking and nose licking were more frequently expressed when dogs were coping with frustration in a social context rather than in an analogous non-social one and did not correlate with cortisol concentrations [22], enforcing the idea of a social communicative intent of those signals (see also [5,17,25]). In another study [26], dogs approached by unfamiliar humans with either a neutral or threatening attitude (TAT: Threatening Approach Test [27–30]) expressed more frequent blinking, nose licking and lip wiping when they didn’t show aggressive behaviours, supporting an appeasement function of those signals [20,22,24,26]. Finally, when exposed to videos of neutral versus threatening conspecifics, dogs expressed blinking, sniffing, yawning, lip wiping and nose licking more frequently towards a neutral dog, suggesting that they may also be exhibited in ambiguous contexts when the behaviour of the upfront social partner is difficult to predict [31].

In several species showing high levels of social tolerance and cooperation, it is known that facial displays can be related to social contagion situations. For example, contagious yawning was found in: gelada baboons (*Theropithecus gelada*) [32]; bonobos (*Pan paniscus*) [33,34]; lions (*Panthera leo*) [35]; hyenas (*Crocuta crocuta*) [36]; spider monkeys (*Ateles geoffroyi*) [37]; African painted dogs (*Lycaon pictus*) [38] and wolves [39]. Similarly, the so-called ‘play face’ (i.e. also known as ‘relaxed open mouth’—ROM), the iconic facial expression related to social play aimed at limiting misunderstandings, is shown by many mammal species and results in an effective metacommunicative signal aimed at sending a playful message [18,40]; ROM found in: slender-tailed meerkats (*Suricata suricatta*) [41]; South American sea lions (*Otaria flavescens*) [42]; sun bears (*Helarctos malayanus*) [43 gelada baboons, 8]; bonobos [44]; Bornean orangutans (*Pongo pygmaeus*) [45]; wolves, and coyotes (*Canis latrans*) [14,46]. Both contagious yawning and play face mimicry are also well-documented in domestic dogs (see [47] for an extensive review).

Some facial expressions seem to carry multiple functions, expressing emotional states, playing a role in social communication [17,48], or satisfying an anatomical need, depending on the context. For example, eye blink is rooted in the evolutionary history of tetrapods [49,50] and protects the eye from damage and prevents the surface of the cornea from drying. In addition, animals use it to change the direction of gaze and show directional responses. Species experiencing predation adjust blink frequency based on group size [51,52], blinking more often in groups where they can rely on others for vigilance, and less when alone to maintain constant environmental awareness (e.g. red deer (*Cervus elaphus*) [53]; olive baboon (*Papio anubis*) [54]; red junglefowl (*Gallus gallus*) [55]). In both human and non-human primates it is known that eye blink, besides protecting the eye, also plays a role in non-verbal social communication [52,56–58]. Humans coordinate the timing of their blinks with their social partners, a phenomenon known as blink entrainment, not expressed by individuals suffering from Autistic Spectrum Disorder (ASD)—for whom one of the main symptoms is impaired social communication [56,59]. Similarly, in non-human primates, blink occurrence increases proportionally to group size [57] and shows temporal coordination between social partners engaged in dyadic interactions [60,61]. Rhesus macaques (*Macaca mulatta*) have shown eye entrainment while watching videos of conspecific social interactions and this implies that synchrony of this facial display is not an automatic imitation of blinking but a basic form of social communication [58]. Similarly, narrowing the eyes seems to be associated with positive emotional communication in wild canids [14], horses (*Equus ferus caballus*) [62], cattle (*Bos taurus*) [63] and cats (*Felis silvestris catus*). Cats slowly blink to communicate a positive emotional state to humans, and tend to respond to blink performed by their caregiver [64]. It has also been observed that cats in rescue shelters showing an increased occurrence of blinking were rehomed faster [65] It is possible that dogs would also engage in blink mimicry due to the alleged function of synchronizing social activity and communicating this behaviour, as previously found in other human and non-human species (in *humans* [56,66]; *non-human primates* [57,58]; *amphibians (Odorrana tormota)* [67]. Considering the aforementioned evidence of the social function of eye blink, the present study aimed to further explore the possibility that blink plays a role in dogs social communication. To assess the specific impact of blinking, dogs were exposed to three types of video stimuli: one showing conspecifics blinking their eyes, another showing them licking their noses, and a control video where the conspecifics were attentive still looking at the camera.

The nose-licking behaviour, whose primary physiological functions are to keep the outer and inner nose surfaces clean [68], cool and moist, and to improve olfactory abilities providing an optimal environment for detecting scents (domestic dog [69]; domestic cat [70,71]) was chosen as facial expression alternative to blink, since it has also been suggested to have social communicative functions in dogs [5,22,26,31], and it is expressed both during positive anticipation [16] and frustration [21]. By including nose licking as an alternative facial expression, the study aimed to see if dogs’ reactions were specifically triggered by the blinking and/or nose-licking behaviours, rather than simply by the face of a still attentive dog. If eye blinking and nose licking have a social synchrony function, we expect them to elicit the same visual signal in the receiver compared to the attentive still condition. Recent research on the emotional perception in dogs has increasingly used paradigms involving video and image exposures [26,72–74]]). Specifically, Mongillo and colleagues [75] demonstrated that dogs are able to recognize a conspecific in videos featuring either a dog or another species (horse/cow).

For a broad comprehension of dogs’ visual communication, the emission of other facial expressions (for example ear positions) that could be elicited by blinking and nose licking were investigated using the DogFACS [13,76], which is an objective coding system based on facial muscles and adapted from the original ‘HumanFACS – Facial Action Coding System’ [77]. The need for a standardized tool was underlined by [78,79] who have shown that even experts and professionals cannot be completely accurate when assessing some emotions (e.g. aggressiveness) and that some phenotypic characteristics, such as a darker colouring, may interfere with the detection of subtle emotional cues by untrained observers when a standardized system is not applied. DogFACS has become a relevant and widespread tool for the understanding of canine behaviour since it relies on objective facial movements shared across different dog breeds [13,17,21,22,80,81]. Given that previous research pointed out that dogs’ facial expressions are triggered by the stress response [82–84], it was also important to consider whether these displays were related to an autonomic nervous system activation. While previous studies have relied on cortisol measurements to assess ANS activation [22,81], the same approach may not effectively capture short-term stress responses. A previous study by Siniscalchi and colleagues [4] showed that dogs’ heart rate (HR) rapidly changes according to the exposure to digital photographs portraying human emotional states (namely fear, anger and happiness). Therefore, in the present study, cardiac activity was monitored through HR and heart rate variability (HRV), which provide a more immediate and nuanced view of autonomic nervous system activation [85–87]. If the projection of facial expressions is associated with an autonomic nervous system activation, then HR should increase while dogs are watching the videos and should return to baseline values just after the exposure.

## 2. Material and methods

### 2.1. Ethical note

All the procedures were approved by the ethical committee of the University of Parma (approval number PROT.N.6/CESA/2022). Owners were informed about the experimental procedure and signed a consent form.

### 2.2. Pilot study

A preliminary pilot study was carried out to ensure methodological validity since, to the best of our knowledge, there are no published studies carried out with videos showing facial signals, without an accompanying vocal signal to trigger dogs’ attention. The aim of this pilot study was to verify that (i) dogs watch the videos, (ii) neither the identity of the three actor dogs, (iii) nor the type of signal exhibited in the video directly affects the time spent watching the videos.

### 2.3. Pilot study—Material and methods

#### 2.3.1. Pilot study—Setup and procedure

The pilot study was conducted at the University of Parma in a 3×4 m experimental room that featured a white projector screen, dark green side panels forming a corridor to allow the maximum dog’s attention on the video stimuli and a leash for the dog attached just below the projector (Seiko Epson H435B) on the back wall. Three cameras (Atlantis F930HD) recorded the dog’s behaviour from different angles, while a chair for the owner and a water bowl for the dog were also provided.

On arrival at the university, the dogs were allowed to explore the experimental room for 5 min to settle in while the owner completed paperwork and fitted the dogs with an H-shaped collar. In the pilot study, each subject was exposed to one blink and one NL video (the order of presentation was counterbalanced between subjects). The tested dogs were given a 5 min break between each video to avoid fatigue and loss of interest. The entire procedure was completed in a single session, lasting approximately 15 min.

#### 2.3.2. Pilot study—Subjects

Twenty-four dogs (which did not participate in the main study) of different breeds, ages (mean = 5.5 y) and sex (13 males and 11 females) were recruited, including 8 shepherd dogs, 9 hunting dogs and 7 dogs of other breeds (such as molosseoids and primitive breeds), details in electronic supplementary material Table S33. Two dogs that never paid attention to the videos were discarded and the sample resulted in 22 dogs (13 males and 9 females).

#### 2.3.3. Pilot study—Video stimuli

**Figure 1.**
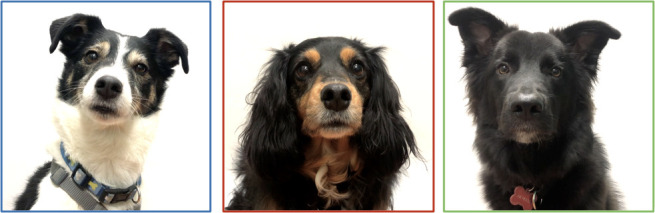
Pictures of the three actor dogs (respectively Dog1, Dog2, Dog3).

As previous studies [22,31] showed that the two behaviours of interest (i.e. blinking and nose licking) are associated with the presence of a human audience, the videos were recorded placing each actor dog in front of a camera and asking him/her to sit keeping the attention towards his/her owner. The actor dogs were of mixed breeds: Dog1—a 6-year-old terrier-like mix with buttoned ears; Dog2—a 5-year-old cocker spaniel mix with floppy ears and Dog3— a 1-year-old border collie mix with almost fully erect ears (figure 1). From the videos recorded, close-ups of the three dogs with a white background were extracted using Final Cut Pro (version 10.6.2, Apple Inc., USA) and merged to have a sequence lasting 12 s with the performance of the facial signal three times for each actor dog, i.e. one nose lick (NL) or blink every 4 s.

**Figure 2.**
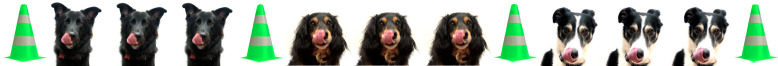
Timeline summarizing a video session. 1 s per green-cone; 12 s per actor dog (Dog3–Dog2–Dog1, NL1).

The final video 40 s each: 36 s featured the three actor dogs performing the target facial signal (NL or B) and 4 s of a green cone accompanied by a ‘beep’ sound, at the beginning, after each actor dog performed the facial signal and at its end (figure 2).

As the attentive still video was not part of the pilot, a total of six different videos were created for both the NL and the B, covering all possible actor dog combinations (table 1). Each subject was tested with one NL and one B video presented in a semi-randomized order and counterbalanced across the sample.

**Table 1.** Summary of the videos created mixing all the possible actor dog combinations for both facial signals nose lick (NL) and blink (B) (NL1 is depicted see figure 2).

order of the actor dogs in videos performing NL or blink			
dog3–dog2–dog1	NL1	dog2–dog3–dog1	B1
dog3–dog1–dog2	NL2	dog1–dog2–dog3	B2
dog2–dog1–dog3	NL3	dog1–dog3–dog2	B3
dog1–dog2–dog3	NL4	dog2–dog3–dog1	B4
dog2–dog3–dog1	NL5	dog3–dog1–dog2	B5
dog1–dog3–dog2	NL6	dog3–dog2–dog1	B6

#### 2.3.4. Pilot study—Behavioural coding

The time spent looking in the direction of the video was coded with BORIS (version 7.13.9, University of Torino, Italy) [88].

#### 2.3.5. Pilot study—Statistical analyses

To assess whether the identity of the actor dogs (Dog1/Dog2/Dog3) and the type of facial signal shown (B/NL) influenced the amount of time the subjects spent looking at videos (dependent variable), a linear mixed model with a one-way interaction between the actor dog identity (hereinafter referred to as ACTOR ID) and the facial signal (hereinafter referred to as SIGNAL) was run with R, version 4.2.3 [89], using ‘lme4’ package, function ‘lmer’ [90]. The model also included sex, age, neutered state and breed type of the dog tested as control predictors, while the subject ID was included as a random effect to account for the repeated observation.

The interaction between the ACTOR ID and the SIGNAL did not significantly influence the time dogs spent looking at the video, therefore, it was removed. We applied Tukey’s post hoc tests to evaluate multiple pairwise comparisons. Confidence intervals (CI) for each pairwise comparison were also calculated. The normality of residuals was checked with QQ plots and all models exhibited a satisfactory distribution of residuals. Results were considered statistically significant if *p* ≤ 0.05.

#### 2.3.6. Pilot study—Results and conclusion

The tested subjects looked at the videos projected for 35.5% of the total time (x̄ = 12.782 ± 8.877). Neither the identity of the actor dog (Dog1/Dog2/Dog3) nor the type of facial signal (NL/B) significantly influenced the time spent looking at the videos during the test (χ^2^ = 1.397, *p* = 0.497) in the interaction model. Similarly, in the no-interaction model, the results show that neither the identity of the actor dogs (χ^2^ = 3.005, *p* = 0.223), nor the type of facial signal (χ^2^ = 1.224, *p* = 0.269), had a statistically significant effect on the time the dogs spent watching the stimuli. Overall, (i) dogs kept a good level of attention towards the videos; (ii) blink and NL were similarly interesting for dogs (this result was not granted as blink is a more subtle and less conspicuous signal than NL); and (iii) the identity of the actor dogs did not influence the dog’s attention.

## 3. Main study

### 3.1. Subjects

Fifty-four dogs, 30 females (24 neutered, 6 intact) and 24 males (10 neutered, 14 intact), aged between 1 and 12 years (mean = 6.06) were tested in a within-subject design study (electronic supplementary material, table S1). Medium to large size (small sizes were not included due to the Polar WearLink® strap minimum length), purebred as well as mix-breed dogs were recruited. Only mesocephalic dogs were included, to control for the influence of morphology on the facial expressions exhibited. Subjects were recruited from the database of our laboratory and adverts on social media and none of them was familiar with the dog actors. The inclusion criteria for the subjects required that dogs had to be awake, with eyes open and maintain head orientation towards the stimulus for at least 4 s within the video projection.

### 3.2. Setup

**Figure 3.**
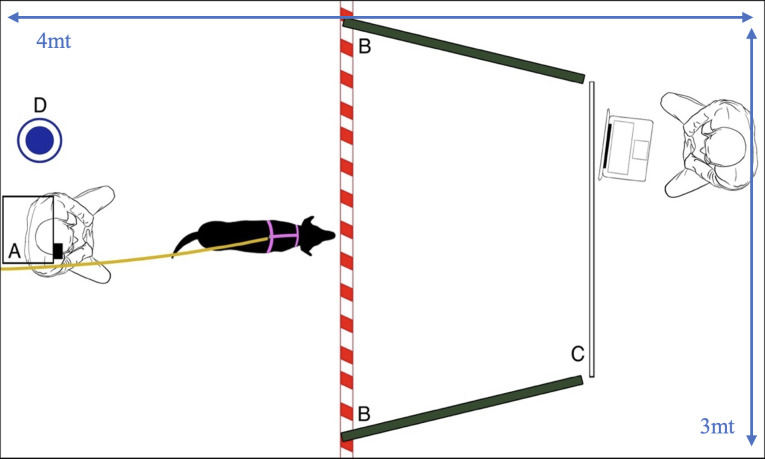
Representation of the experimental setup, (A) projector, (B) dark green panels and white dashed line on the floor, visually delimiting the space inaccessible for the dog, (C) projector screen, (D) water bowl.

The experiments took place in the same laboratory as the pilot: a 3 × 4 m room, partitioned by a wall-like apparatus with an attached white projector screen. Placed behind this apparatus was a computer and a chair for the experimenter. On either side of the projector screen, dark green panels (1.50×1.50 m) covered the walls, forming a corridor to help the dog focus on the video stimuli. The projector (H435B, Seiko Epson Corporation, Japan) was mounted 1.65 metres high and 3 metres from the back wall, facing a white screen. A leash for the dog was affixed to the wall below the projector. Three cameras (Atlantis F930HD, ATL S.r.l., Italy) were in the experimental room. Two cameras were placed to the left and right of the subject, with a third in front of the projector screen. A chair for the owner was below the projector, and a water bowl was always available (figure 3).

### 3.3. Video stimuli

During the test dogs were exposed to three video stimuli:

- ‘Blink’
- ‘Nose lick’
- ‘Attentive still’ (control condition)

**Figure 4.**
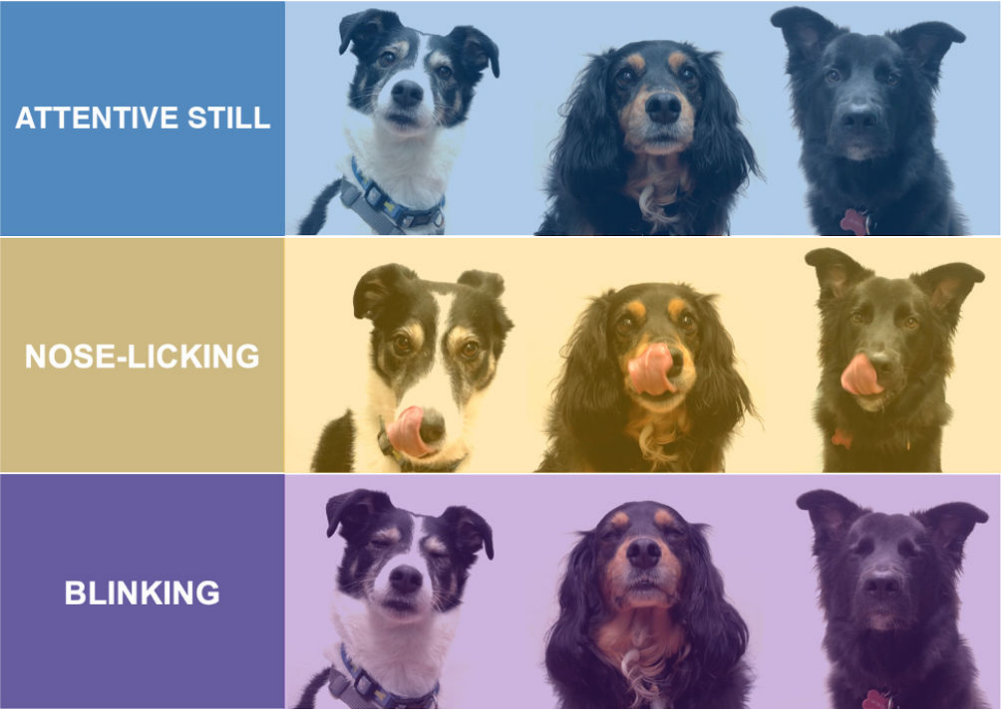
Frames of the videos showing attentive still, NL and B (respectively Actor Dog1, Actor Dog2, Actor Dog3).

Videos with the three actor dogs performing B or NL were made as detailed in the pilot study. For the main study, there was added an attentive still-looking (control) video during which each actor dog stayed still, looking at the camera, without performing any facial expression (figure 4). The attentive still-looking (i.e. ears forward with mouth closed and fixed gaze) of the actor dogs was induced by a food or toy reward held by the owner in front of the dog. At the end of each video, there was also an added 30 s of black screen for the purpose of control for eventual carry-over effect in cardiac activity and another final green cone with the ‘beep’. Thus, the final Video 71 s each: 36 s of actor dogs followed by a 30 s black screen and 5 s for the green cone accompanied by a ‘beep’ sound to increase engagement and attention towards the screen at the beginning, after each performance and at the end of the video. A total of six videos were edited for NL, B and attentive still, covering all possible combinations of actor dog identity.

### 3.4. Experimental procedure

The experimental procedure was similar to the one described above for the pilot study. After arriving at the laboratory in the university, the dogs were given five minutes to explore the room freely. This allowed them to become habituated to the new environment and the experimenter. In the meantime, the owners completed a privacy form and provided essential information (i.e. name, breed, age, sex, castration status) about their dogs. The owners were then instructed to fit their dogs with a standardized H-shaped harness while the experimenter applied the Polar Belt with electrode gel around the dog’s chest. Dogs were given a further 10 min to adapt to the harness and belt.

No signs of distress (e.g. fear, excessive door orientation or hostility towards the unfamiliar experimenter) were observed, and all dogs adapted well to the harness and environment. Following this period, dogs were leashed and the experimenter activated the Polar belt to measure the dog’s baseline heart rate. After the 5 min baseline, the experimenter left the room while another experimenter, who was well-hidden behind the apparatus and unknown to the dog, started the video projection and recording. During the test phase, owners were instructed not to interact with their dogs to avoid the expression of unintentional cues, while sitting for the whole test duration on the chair against the back wall (shown in figure 3).

Each subject was exposed to attentive still, one B and one NL video: the order of the video presentation was counterbalanced between subjects. Tested dogs were given a 5 min break between each video to avoid fatigue and loss of interest, and to allow their heart rate to return to baseline levels.

The decision not to include a pre-test period of habituation in the experimental environment involving different tasks with toys or food was made in order to keep the focus on the dogs' spontaneous reactions to the videos of conspecifics, avoiding the introduction of tasks that could create expectations or distractions and to prevent fatigue. The entire procedure was completed in a single session, lasting approximately 28 min (table 2). The room was cleaned up after every test session.

**Table 2.** Complete timeline of the entire experiment.

session timeline	
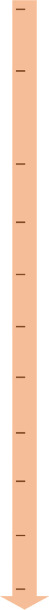	initial habituation: the dogs were free to explore the test room for 5 min.
	Polar Belt and harness habituation: Owners put on their dog the H-shaped harness, followed by the application of the Polar belt. This on-leash lasted 10 min and helped the dogs getting used to the harnesses.
	FIRST VIDEO SESSION
	cardiac baseline recording: on leash, it followed a 5 min period to record cardiac activity to extract a baseline value.
	first video projection: the video lasting 71 s was one of the following: NL, blinking or attentive still.
	SECOND VIDEO SESSION
	cardiac baseline recording: on leash, it followed a 5 min period to record cardiac activity to extract a baseline value.
	second video projection: a second video was presented, showing one of the two remaining videos.
	THIRD VIDEO SESSION
	cardiac baseline recording: on leash, it followed a 5 min period to record cardiac activity to extract a baseline value.
	third video projection: the last of the three videos was presented.

## 4. Behavioural coding

All the videos were coded using BORIS. The two ethograms consisted of one with selected facial expressions and actions from the DogFACS manual. Following the analysis, all behaviours that were expressed by fewer than 6 dogs (10% of the sample) were excluded (table 3). Both coders, CC and RF, were DogFACS certified coders, and inter-coder reliability was assessed using interclass correlations (ICCs [91]) on 20% of the videos (ICC from 0.71 to 0.92).

**Table 3.** Behaviours that were expressed by more than 10% of the tested subjects (at least six dogs). For the complete ethogram and descriptions see the Supplementary Material (electronic supplementary material, tables S2 and S3).

		dogFACS’ codes
**dogFACS** **>10%**	blink	AU145
	lip wipe (right / left)	AD37-R / AD37-L
	nose lick	AD137
	sniffing (around/door/owner)	AD40-A / AD40-D / AD40-O
	panting	AD126
	ears forward	EAD101
	ears adductor	EAD102
	ears flattener	EAD103
	ears rotator (both / right / left)	EAD104/EAD104 R / EAD104-L
	ears downward	EAD105
	sclera	
**general behaviours** **>10%**	head and body orientation (door/owner/apparatus/stimulus/ elsewhere in the room)	
	paw lifting	
	autogrooming	
	looking away	
	contact with the owner	

### 4.1. Cardiac activity

Heart rate data were collected using a Polar® RS800CX HR monitor (Polar® Electro, Finland). The Polar WearLink® strap was positioned around the dog thorax, its size was adjusted to provide a tight but comfortable fit, and it was kept in position using harnesses dogs were already used to. Farmacare ultrasound transmission gel (Farmacare, Italy) was applied to the two electrodes of the Polar WearLink® strap. The electrodes were positioned over the right and left axillary regions. The Polar® watch computer was fixed dorsally to the WearLink strap; therefore, it was always within 30 cm of the Polar WearLink® strap. It was set on the R-R interval-recording mode and data collection lasted for the whole duration of the experiment. R-R interval data were analyzed using Kubios HRV Scientific (version 4.1.0, Kubios Oy, Finland [92]). Prior to analyses, artefacts were removed using Kubios HRV scientific’s inbuilt artefact correction feature. The artefact tolerated was not >5% on the total length of the recording, for each dog. Mean heart rate (HR, beats per minute) and the following time-domain HRV parameters were calculated for each experimental phase: root mean square of the standard deviation of R-R intervals (RMSSD, ms), standard deviation of R-R intervals (SDNN, ms), SDNN/RMSSD ratio and the percentage of number of pairs of successive R-R intervals that differed by more than 50 ms over the total number of R-R intervals (pNN50, %).

Dogs that occurred in an anomalous cardiac tracing were excluded by the analysis; therefore, only 23 dogs out of 54 were analyzed (42.59%). A Polar® malfunctioning, resulting in a fixed data acquisition problem approximately 25 min after activation, resulted in a loss of cardiac activity in the dogs during the third video projection. Therefore, only the cardiac parameters of the dogs that were recorded during the baseline, and the first and second video sessions were included.

### 4.2. Statistical analyses

A linear model was run using R (v. 4.2.3 [89]), to examine the interaction between the duration ‘looking at the video’, dependent variable, and the two main test predictors: SIGNAL (NL, B or attentive still—named ‘control’ in the analyses) and ACTOR ID. The order of the video presentation, age, sex, neuter status and breed type of the tested dogs were entered as control predictors. Subjects ID was introduced as a random effect. Finally, since the interaction between the two test predictors was not significant, the model was simplified removing the interaction between SIGNAL and ACTOR ID. Both analyses showed *p*-values above threshold (*p* > 0.05) for each contrast (function: ‘TukeyHSD’, package: ‘emmeans’ [93]). The CIs were also calculated for every pairwise comparison (R generic function ‘confint’). Following this finding, we performed the next analyses merging the durations and frequencies of the behaviours displayed by the same subject when exposed to different actor dogs in the same SIGNAL video. A linear mixed model was used for behaviours coded as ‘duration’, whereas behaviours coded as ‘frequency’ were analyzed using generalized linear mixed models with Poisson error distribution (function ‘glmmTMB’, package ‘glmmTMB’ [94]). One model was run for each behaviour (dependent variable) displayed by the dog during the test. To assess for potential effects of individual variation, control predictors such as sex, age, breed type and neuter status were additionally included. Subject ID was included as random effect.

The sequence of cardiac recording of each video session was divided into three phases as follows: the final 30 s of the 5 m baseline recording was considered as BASELINE phase, the 40 s of video projection with the actor dogs was considered as VIDEO phase and the 30 s of black screen was considered as POST phase. This allowed us to evaluate potential differences between the baseline and the video projection, and to assess potential carry-over effect of a stimuli-related cardiac activation.

Cardiac activity was analyzed using linear mixed models with the cardiac parameter of interest as the dependent variable (HR, SDNN, RMSSD, PNN50, ratio SDNN/RMSSD), the test condition as test predictor and the sex, age, neutered state and breed type of the dog tested as control predictors, and Subject ID as random effect. A general model was fitted including first and second video (cardiac data of the third video were lost as mentioned above) to ensure that dogs after the first video returned to cardiac baseline values (baseline 1, video 1, post 1, baseline 2, video 2, post 2). Since no differences emerged among phases of first and second video for any of the cardiac variables considered (baseline 1 versus baseline 2; video 1 versus video 2; post 1 versus post 2) it was decided to run two separate repeated ANOVA for the video session 1 and the video session 2 (‘lmer’ function, package ‘lme4’ [90]) with the analyzed cardiac parameter as the dependent variable and the categorical variable SIGNAL was also employed to control for possible differences among phases and specific facial signal (Baseline/Nose Lick/Blink/Attentive Still/Black screen). This model adjusted for subject grouping, treating subjects as random effects to accommodate potential similarities among observations within the same ‘Subject ID’ group. In addition, the individual characteristics age, sex, neutered state and breed type were included in all models as predictors. The ‘TukeyHSD’ function was used to examine pairwise differences. This post hoc analysis provided a detailed insight into the specific contrasts between the factor SIGNAL. Results were considered statistically significant if *p* ≤ 0.05. For further details about all the analyses, see electronic supplementary material, table S4 to S3.

## 5. Results

Behavioural analyses were performed on 47 dogs, 26 females and 21 males, since 7 subjects did not meet the inclusion criteria.

### 5.1. DogFACS variables

**Figure 5.**
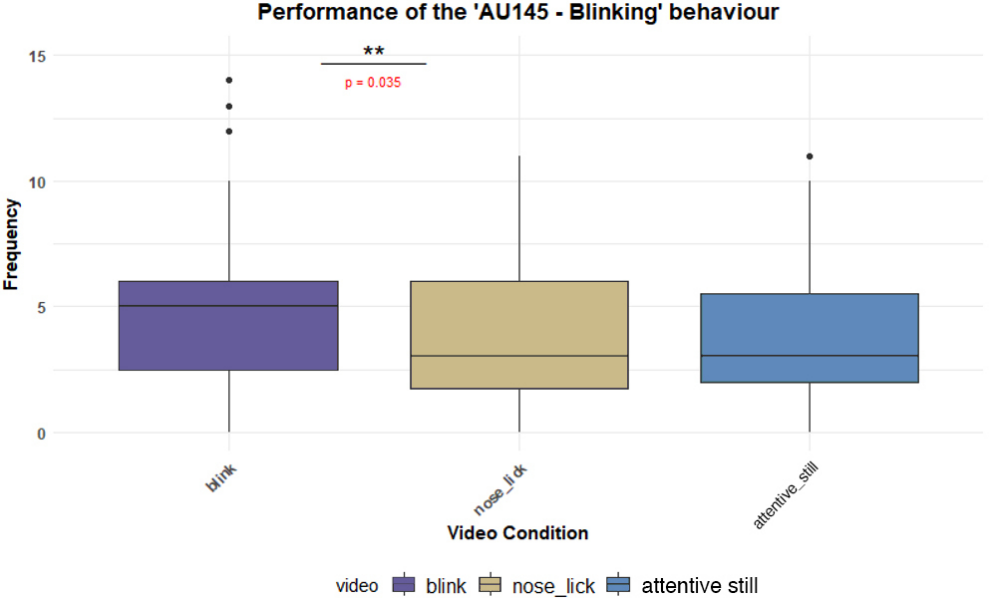
Frequency of the ‘AU145–- Blinking’ behaviour in the different video conditions

The results indicated that the ‘Blink – AU145’ behaviour occurred significantly more frequently in response to the blink signal compared to the NL signal (Es: 0.250 ± 0.101, *p* = 0.035 95% CI [0.99, 1.47], figure 5). A similar trend was observed when it was compared to the attentive still video (Es: 0.220 ± 0.099, *p* = 0.071 95% CI [1.22, 1.68]). Conversely, the expression of the ‘Nose Lick – AD137’ behaviour did not differ significantly across any of the signals presented. The ‘Sclera’ display was prolonged during the projection of the NL video compared to the attentive still video (Es: −1.491 ± 0.582, *p* = 0.032 95% CI [−2.88, −0.10]). However, no significant differences were found in the other comparisons (blink versus attentive still; blink versus NL). No significant differences were observed in any other DogFACS behaviours among the signal treatments (See Supplementary Materials for detailed results, electronic supplementary material, table S4 to S17). Regarding sex differences, male dogs exhibited the ‘Nose Lick – AD137’ behaviour more frequently than females, independently from the signal (Es: 0.803 ± 0.291, *p* = 0.006 95% CI [−0.06, 0.13]). Additionally, hunting dogs maintained the ‘Ears adductor – EAD102’ position longer than shepherd dogs (Es: 7.70 ± 2.18, *p* = 0.0029 95% CI [2.40, 13.01], whereas they held the ‘Ears rotator – EAD104’ position for a shorter duration compared to shepherds (Es: −9.57 ± 2.22 *p* = 0.0003 95% CI [−15.0, −4.16]). Age influenced the ‘Sclera’ duration (Es: 0.264 ± 0.113, *p* = 0.024 95% CI [0.05, 0.48]). No other DogFACS behaviours were associated with sex, age, neuter status or breed type.

### 5.2. General behaviours

The average time dogs spent looking at the videos was consistent with the pilot study findings (x̄ = 12.022 ± 11.043 s out of 36 s). None of the general behaviours analyzed were found to be significantly related to any of the predictor variables (see the Supplementary Materials for the detailed results).

### 5.3. Cardiac activity

The repeated-measures ANOVA did not reveal any significant differences in cardiac parameter among the three signals projected (i.e. ‘blink’, ‘nose lick’ and ‘attentive still’). However, significant variations in SDNN intervals were observed across different video phases. SDNN values increased from baseline to the black screen phase (hereinafter referred to as ‘post’). Post hoc analysis indicated a significant difference between baseline and post during the first video session (Es: −22.30 ± 7.66, *p*‐value = 0.0425 95% CI [−44.10, −0.507]) and again during the second video session (Es: −24.50 ± 7.12, *p*‐value = 0.010 95% CI [−44.65, −4.34]). A similar trend was observed for RMSSD, with significant differences noted during the second video session between baseline and post (Es: −43.15 ± 13, *p*‐value = 0.014 95% CI [−80.0, −6.34]). Additionally, pNN50 values increased during video sessions, significantly increasing only between baseline and post of the second video session (Es: −10.72 ± 3.60, *p*‐value = 0.038 95% CI [−21.06, −0.39]). See Supplementary Materials for graphics and detailed results (electronic supplementary material, table S18 to 27 & electronic supplementary material, figure S5 to S14).

## 6. Discussion

The aim of the present research was to explore the possibility that blink plays a role in dogs' social behaviour, as suggested by previous studies [22,31]. To assess the specific impact of blinking, dogs were exposed to three types of video stimuli: one showing conspecifics blinking their eyes, another showing them licking their noses, and one where the conspecifics were attentive still-looking towards the camera. The main finding of the present study is that the blink video elicited experimental subjects to express blink display more frequently (Blinking—AU145 for DogFACS) compared to the NL video and the attentive still video (although this difference is statistically significant only between blink and NL video). Eye blinking seems to support non-verbal social communication in primates, as it has been shown that humans synchronize blinks with social partners [56] and non-human primates, like rhesus macaques, also coordinate blinks during interactions, suggesting that its function is more than just eye protection [58]. In dogs, blinking has been considered as an appeasement behaviour [4] used to express non-aggressive intentions towards conspecifics, consequently its synchronization within a group could be a sign of a mutual communication or understandings. Reciprocal blinking in dogs might help to facilitate conspecific social bonds, cope with frustration and communicate non-aggressive intentions, as already shown in the interspecific context with humans. In fact, previous findings have shown that dogs exhibit this behaviour more frequently when experiencing frustration, whether in non-social [21] or social situations [22]. Furthermore, a recent study from Koyasu and colleagues [95] highlighted that during dyadic interactions between the owner and its pet (dog and/or cat), a reciprocal blink synchronization occurred, with humans and pets blinking immediately after their social partner. To the best of our knowledge present results are the first evidence that reciprocal blinking occurs between domestic dogs suggesting a role also in intraspecific communication. Future studies are needed to further investigate the contexts in which it occurs and the timing of the response to verify if it can be a case of mimicry. This specific response pattern was instead not observed for the NL, as the nose licking video did not elicit an increase in the nose licking responses and its frequency remained consistent across all conditions, suggesting it likely does not hold the same function of the blink. The feedback for the NL signal was instead the increased display of the sclera, also known as ‘whale eyes’ [96,97] a behaviour previously observed both in stressful agonistic situations (Handleman 2012) and in fear-related contexts [98]. It is possible that the nose licking behaviour in the videos may have been perceived/interpreted by the subjects as a signal related to agonistic contexts, eliciting the showing of the sclera (see discussion below for further results about the nose licking behaviour). Future research should also investigate the possible relationship between sclera visibility and age as, to our knowledge, no existing study has assessed this correlation.

Other results showed differences related to dogs’ sex and breed. NL behaviour was more frequent in male than female dogs. This trend mirrors previous findings, where male dogs exhibited more lip wiping behaviour [81]. It has been previously reported that male dogs express behaviours of aggression, territorial defence and boldness (both towards conspecific and interspecific individuals) more than females [99–101]. Male dogs, especially those of higher social rank, often display behaviours influenced by intra-sexual competition, female preferences and pack dynamics [102,103]. While nose and lip licking behaviours have been previously associated with appeasement strategies in dogs [5,23,30,104], it is possible that these behaviours might also serve as threat-related signals under conflict contexts. The display of showing the sclera discussed above could support an agonistic function of the NL signal that deserve further study.

Finally, two Ear Action Descriptors (EADs) analyzed, ‘Ears Adductor - EAD102’ and ‘Ears Rotator - EAD104’ were expressed significantly more by hunters and shepherd dog types, respectively. A similar result was also found by Pedretti *et al*. [22], with hunting dogs keeping their floppy ears more ‘downward – EAD105’ than shepherd dogs, whereas shepherd dogs with pointed ears kept them more ‘flattened – EAD103’. This difference is likely due to the anatomical characteristics of the breeds we considered, but it is also possible that it is related to other specific behaviours and motor patterns that in shepherds are traditionally directed towards the livestock and seem to be strictly heritable [105]. We acknowledge that selection for specific traits (e.g. attention to visual stimuli in shepherd dogs) could have impacted on the attention and relevance of the visual signals. However, no difference was found in the duration of the attention towards the videos in shepherds versus hunting types, suggesting no influence of the factor ‘breed type’ to the attention span of the dogs to the stimuli.

Further studies will be needed to specifically investigate how sex and/or breed types have an impact on the emittance of some behavioural displays.

Dogs’ heart rate was stable across the phases of the test and no differences emerged among HR values recorded during the three different video SIGNALS (i.e. blink, NL, attentive still videos). Three out the four time-domain parameters of HRV computed increased across the phases: SDNN, RMSSD and pNN50 were always higher in the post test rather than in the baseline phase independently of the SIGNAL projected. Considering that SDNN, RMSSD and pNN50 are positively correlated with the parasympathetic branch (Task Force of the European Society of [106–108]) the overall pattern of cardiac results suggest that the video test context was perceived as a non-stressing situation and the behaviours expressed by the experimental subjects are not merely related to an increase of the arousal state.

## 7. Conclusions

In conclusion, this study investigating the feedback of domestic dogs to relevant conspecifics’ facial visual cues such as blink and NL further support the hypothesis that these facial expressions play a relevant role in dogs’ social communication and that eye blink could be related to social contagion. The present results pave the way for further investigation of blink synchronization, as this aspect was not directly addressed in the present study. Future research should also explore the effects of these signals in modulating various social interactions, such as agonistic and affiliative contexts.

## Data Availability

Data and relevant code for this research work are stored in GitHub: [https://github.com/claire-cnr/ifyoublinkatmeillblinkback_DATASET.git] and have been archived within the Zenodo repository [109]. Supplementary material is available online [110].
